# A filter-based cross-sectional analysis of an HIV-positive, HAART-treated cohort in rural Burundi: pharmacokinetics, pharmacogenetics and viral load

**DOI:** 10.1186/1758-2652-13-S4-P179

**Published:** 2010-11-08

**Authors:** A Calcagno, MG Milia, A D'Avolio, P Ndayishimiyae, P Dusabimana, S Bonora, J Cusato, M Simiele, R Rostagno, M Siccardi, S Audagnotto, V Ghisetti, G Di Perri

**Affiliations:** 1 University of Torino, Department of Infectious Diseases, Torino, Italy; 2 Ospedale Amedeo di Savoia, ASLTO2, Microbiology and Virology Laboratory, Torino, Italy; 3 University of Torino, Pharmacokinetics and Pharmacogenetics Laboratory, Torino, Italy; 4 Hopital de Kiremba, Kiremba, Ngozi, Burundi

## Background

In Burundi Triomune® is the first-line HIV treatment and it is estimated to reach 30% of those in need, but efficacy monitoring does not rely on viral load (VL) quantification, due to cost and technical limitations. Furthermore nevirapine (NVP) is known to have a highly variable pharmacokinetics (PK) and pharmacogenetics (PG), but no data are currently available in this population.

## Purpose of the study

To study virological outcome, PK and PG of nevirapine-based HAART in Burundi, by relying on previously validated alternative tools for samples collection.

## Materials and methods

A cross-sectional analysis was performed at the rural hospital of Kiremba, northern Burundi. All patients on HAART (>6 months) presenting for care and willing to participate were enrolled. After sample collection, whole blood (50 µL) was spotted on Whatman 903 Cards (Dried Blood Spot, DBS); afterwards plasma (100 µL, after centrifugation) was spotted on glass paper filter (dried plasma spots, DPS). DBS were used for VL testing (NucliSENS EasyQ HIV-1 v2.0) and PG analysis (516G>T and 983C>T SNPs in CYP2B6, 3435C>T and 1236 C>T in MDR1). A validated HPLC method was used to measure NNRTIs concentrations on DPS.

## Results

239 patients (68.2% female) were enrolled; mean (±SD) age and BMI were respectively 37.9 years (±10) and 20.7 Kg/m^2^ (±2.8). The majority of them (90.8%) were in WHO stage 3 and last CD4+ cell count was 543 cell/mm^3^ (±345). 237 were on first line treatment (220 on NVP and 17 on EFV) and 2 (0.8%) on second line (LPV/r). Mean time on treatment was 25.7 months (±13.7) and it correlated to the last CD4+ count (Pearson 0.23, p=0.001). 43 (18%) had a detectable viral load with 14 patients (5.8%) having more than 800 copies/ml. Nevirapine and efavirenz Ctrough were 7727 ng/ml (± 3796) and 4027 ng/ml (± 3041). CYP2B6 mutated SNPs were common (48,5% in 516G>T, 13,6% in 983C>T) and associated to increasing exposure (p=0.01 and p=0.02). A higher proportion of patients (95.6% vs. 88.5%, p>0.05) had viral loads below 800 copies/ml in the higher range of NVP concentration (Ctrough >4300 ng/ml).

## Conclusions

DPS and DBS showed to be useful tools to collect and transport samples from a rural area of Burundi. Even with the limit of a cross-sectional analysis a high effectiveness was noted, showing 82% of patients with undetectable VL at a mean of 2 years since start of treatment. High NVP plasma concentrations along with favorable genetic profile could partially explain these results.

